# Pharmacokinetic modelling as a tool to assess TB treatment adherence: application to the REMEMBER study

**DOI:** 10.5588/ijtldopen.25.0467

**Published:** 2026-02-11

**Authors:** N. Abdelgawad, M. Chirehwa, H. McIlleron, C. Kanyama, N. Mwelase, A. Naidoo, J. Kumwenda, M. Nyirenda, W.P. Samaneka, J.R. Lama, L. Mohapi, V. Mave, V.G. Veloso, J. Valencia, A. Gupta, M.C. Hosseinipour, K. Scarsi, P. Denti

**Affiliations:** 1 Division of Clinical Pharmacology, Department of Medicine, University of Cape Town, Cape Town, South Africa;; 2 University of North Carolina Project–Malawi, Kamuzu Central Hospital, Lilongwe, Malawi;; 3 Clinical HIV Research Unit, University of Witwatersrand, Johannesburg, South Africa;; 4 Center for the AIDS Program of Research in South Africa (CAPRISA), University of KwaZulu-Natal Nelson R. Mandela School of Medicine, Durban, South Africa;; 5 Centre for the AIDS Programme of Research in South Africa (CAPRISA), South African Medical Research Council (SAMRC)-CAPRISA-TB-HIV Pathogenesis and Treatment Research Unit, University of KwaZulu-Natal Nelson R. Mandela School of Medicine, Durban, South Africa;; 6 Johns Hopkins Research Project-Kamuzu University of Health Sciences, Blantyre, Malawi;; 7 University of Zimbabwe Clinical Trials Research Centre, Harare, Zimbabwe;; 8 Asociación Civil Impacta Salud y Educación, Lima, Peru;; 9 Perinatal HIV Research Unit (PHRU), University of the Witwatersrand, Johannesburg, South Africa;; 10 Byramjee-Jeejeebhoy Government Medical College-Johns Hopkins University Clinical Research Site, Pune, India;; 11 Center for Infectious Diseases in India, Johns Hopkins India, Pune, India;; 12 Fundação Oswaldo Cruz, Instituto Nacional de Infectologia Evandro Chagas, Rio de Janeiro, Brazil;; 13 John Hopkins University School of Medicine, Baltimore, MD, USA;; 14 University of North Carolina School of Medicine, Chapel Hill, NC, USA;; 15 Department of Pharmacy Practice and Science, College of Pharmacy, University of Nebraska Medical Center, Omaha, NE, USA.

**Keywords:** tuberculosis, TBI, pharmacometrics, pharmacokinetic modelling, adherence, TPT

## Abstract

**BACKGROUND:**

The REMEMBER (A5274) study found that the four-drug TB preventive regimen did not reduce mortality compared to isoniazid-only, raising adherence concerns. Using drug measurements and pharmacometrics, we assessed adherence in the four-drug arm by comparing participants who developed TB (cases) to those who did not (controls).

**METHODS:**

Using a 1:4 matched case-control design, we analysed stored blood samples at weeks 2, 4, and 8 since treatment start. Rifampicin and pyrazinamide were measured, and adherence was assessed using two thresholds: i) lower limit of quantification (LLOQ) and ii) personalised thresholds derived from pharmacokinetic simulations. Population pharmacokinetic models and Monte Carlo simulations were used to predict individualised thresholds assuming 100% adherence. Conditional logistic regression compared non-adherence between cases and controls.

**RESULTS:**

Among 28 cases and 112 controls, the proportion of samples <LLOQ was 52% (cases) versus 45% (controls) for rifampicin and 20% (cases) versus 14% (controls) for pyrazinamide. Non-adherence was significantly higher in cases compared to controls for two pyrazinamide metrics: the week 4 LLOQ (*P* = 0.050) and the week 2 2.5th percentile personalised threshold (*P* = 0.023).

**CONCLUSION:**

Poor adherence may have contributed to TB incidence in REMEMBER. While not definitive, personalised thresholds from model-based simulations remain useful for adherence assessment.

Approximately one-quarter of the world’s population is estimated to be infected with *Mycobacterium tuberculosis* and ∼5%–10% of those infected will develop active TB.^1^ Immunocompromised individuals, especially people living with HIV (PLWH), are at high risk of developing active TB.^2^ While the historical standard-of-care for TB infection has been a 6- or 9-month regimen of daily isoniazid, several shorter alternative regimens are now available, including 3-month weekly of rifapentine and isoniazid (3HP), 3-month daily of isoniazid and rifampicin (3HR), 1-month daily of rifapentine and isoniazid (1HP), and 4 months of daily rifampicin (4R).^3^ The ‘Reducing Early Mortality and Early Morbidity by Empiric Tuberculosis Treatment Regimens' (REMEMBER) (AIDS Clinical Trials Group [ACTG] A5274) study was a multi-country randomised clinical study in HIV-positive outpatients to compare mortality between the four-drug (empirical) and isoniazid preventive TB therapy. Counterintuitively, the TB incidence rate was significantly higher in the four-drug arm,^4^ prompting questions about whether poor adherence was a contributing factor. Poor medication adherence is often linked to high pill burden, long duration, adverse effects, or absence of symptoms,^5–8^ all of which can diminish adherence to TB preventive therapy (TPT). Adherence can be assessed by indirect (self-reports and pill counts)^9^ or direct (observing the patient taking medication or measuring drug concentrations in biological samples) methods. Therapeutic drug monitoring combined with modelling and simulation-based approaches can be a valuable tool for assessing patient adherence. Moreover, pharmacokinetic models could be combined with spot checks to ascertain whether drug concentrations are within an expected, personalised range. This approach is becoming increasingly accessible due to the development of simple, low-cost point-of-care assays.

Among the four drugs, pyrazinamide and rifampicin are amenable to pharmacokinetic modelling. Upon oral administration, pyrazinamide is rapidly and completely absorbed with an elimination half-life of ∼9 h.^10^ Rifampicin is well-absorbed and induces several drug-metabolising enzymes, resulting in autoinduction. Its clearance doubled after ∼2 weeks of daily administration. Therefore, steady-state concentrations are reached after ∼2 weeks^11^ and for the 300 mg dose, the half-life is ∼2.5 h.^12^ Considering that individuals metabolise drugs differently based on their body size, both drugs are dosed based on weight to ensure adequate drug exposure. Weight-based dosing helps to account for variations in drug distribution, metabolism, and elimination among patients of different weights.

Here, we explore the use of pharmacometric modelling and simulation to assess and compare TPT adherence between participants who developed TB (cases) and those who did not (controls) within the four-drug arm in REMEMBER.

## METHODS

In this sub-study (NWCS440), participants from the four-drug (RHZE) arm who developed TB by week 48 (cases) were matched with participants who did not (controls) by weight and sex in the ratio of 1:4. Further details can be found in the Supplementary Data.

### Sample collection

Opportunistic plasma samples were used, as the study was not originally designed for pharmacokinetic analysis. One blood sample was collected per visit – at weeks 2, 4, and 8. While the times of sample collection were recorded, the time of the last dose was neither recorded by the staff nor reported by the participants. Samples collected during the visit are assumed to be ∼24 h post-dose.

### Methods for identifying medication non-adherence

At weeks 2, 4, and 8, each participant’s adherence status was assessed based on their observed drug concentration relative to a predefined threshold. If the observed concentration was below the threshold, the participant was classified as non-adherent. However, a concentration above the threshold does not definitively confirm adherence; rather, it indicates that non-adherence cannot be conclusively ruled out. Participants were asked if they had missed yesterday’s dose at each visit, and this information was recorded in the case report form. The concentration-based assessment was applied to pyrazinamide and rifampicin, and at each visit separately. We used two different methods to define thresholds for assigning the adherence outcome, as shown below.

1. Using the following lower limit of quantification (<LLOQ) as the threshold.
  The first method uses the LLOQ of each drug’s assay as the threshold for adherence. In this analysis, LLOQ was 0.203 mg/L for pyrazinamide and 0.075 mg/L for rifampicin. If the observed drug concentration is ≤LLOQ, the outcome is considered non-adherent; if it is above the LLOQ, adherence is not confirmed.
2. Using personalised thresholds from model-based simulations.

Population pharmacokinetic modelling was used to simulate and propose individualised threshold values for pyrazinamide and rifampicin for each participant. We used previously developed and published models for pyrazinamide^13^ and rifampicin^11^ from the TB HAART study, a study in PLWH with smear-positive pulmonary TB. Both models account for the main sources of variability in pyrazinamide and rifampicin pharmacokinetics in patients with both TB and HIV at different levels of immunosuppression. Both models were one-compartment disposition models with transit compartments to model absorption. A key feature of both is that disposition parameters were allometrically scaled for body size using fat-free mass (calculated using weight, height, and sex).^14^ Pyrazinamide exhibited first-order elimination, while the rifampicin model captured its clearance saturation and autoinduction, which are dependent on dose and treatment duration. The inclusion of these structural covariates, along with stochastic variability on clearance, bioavailability, absorption rate constant, and absorption mean transit time, ensured incorporation of the main drivers of between-subject variability (BSV). These models were used in a Monte Carlo simulation to generate the individualised ranges^15,16^ within which the observed drug concentrations are expected to be at 24 and 48 h after the last dose if steady-state is assumed (i.e., full induction) and with 100% treatment adherence. Although the drug is meant to be taken every 24 h, we also simulated drug concentrations at 48 h. This allows for flexibility in case a participant did not take their dose on time, exactly 24 h after the last dose. In other words, if there was a delay in taking the most recent dose, the actual time since the last dose may be longer than 24 h. This 48 h threshold assumes that the last dose taken could have been more than 24 h ago but no longer than 48 h.

For each patient, visit, and drug (i.e., using the dose they received and their value of fat-free mass), the model simulated n = 500 values. Based on the resulting distribution, two personalised threshold values were selected: the 2.5th and 5th percentiles for that participant/timepoint. A measured concentration falling below this value means there is less than a 2.5% or 5% chance of observing such a low concentration if the patient had truly been adherent. If the observed drug concentration in a participant is below the personalised threshold determined through simulations, the outcome for that visit is classified as ‘non-adherent’.

### Statistical analyses

The conditional logistic regression test, which is specifically for matched case–control study designs, was used to compare the odds ratio of being non-adherent between the cases and the controls, adjusting for weight and sex. The test also ‘conditions’ or stratifies the analysis based on the matched groups. By performing the analysis within each matched set, the model inherently controls for the powerful confounding effects of all the variables used for matching. Conditional logistic regression was performed separately for each visit (weeks 2, 4, and 8) and timepoint (24 and 48 h) combination. A *P* value equal to or lower than 0.05 was chosen as significant for the increase in odds. Details on drug quantification and software are in the Supplementary Data.

### Ethical statement

Sites obtained ethical approval from local ethics committees. All participants provided written informed consent.

## RESULTS

The study group comprised a total of 140 participants (28 cases matched with 112 controls). Table 1 shows the participants’ characteristics. One case was missing the sample for week 2, two cases were missing the week 4 sample, and two cases were missing their week 8 samples, resulting in a total of 415 observations per drug. For a total of 29 participants, the week 8 visit occurred more than 8 weeks (between 57 and 64 days) from first treatment dose date. Of these, 7 were in the case group and 22 in the control group. A total of 13 participant-visit occurrences (four cases and nine controls) reported missing yesterday’s dose.

**Table 1.** Participant characteristics.

	Median (min–max) or n (%)	
	Cases (n = 28)	Controls (n = 112)
Men	14 (50%)	56 (50%)
Age (years)	36 (25–55)	36 (18–58)
Weight (kg)	56 (34–81)	56 (30–81)
Height (m)	1.63 (1.48–1.80)	1.65 (1.49–1.89)
Fat-free mass (kg)	41.2 (29.9–55.6)	41.9 (29.5–57.7)

### Adherence results

**Figure 1.**
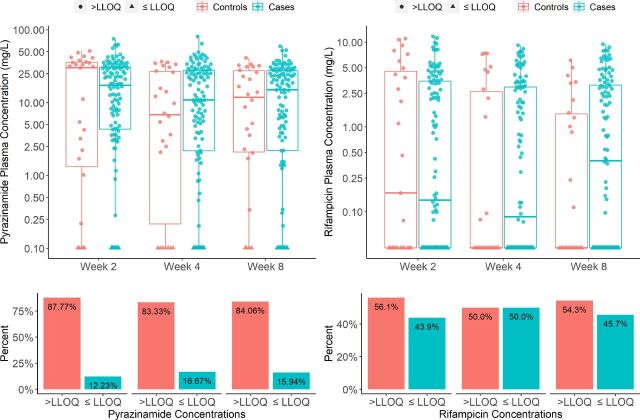
Boxplots of pyrazinamide and rifampicin concentrations stratified by visit week. The lower panel shows the proportion of samples that are below or equal the lower limit of quantification (LLOQ).

1. Using the following limit of quantification (<LLOQ) as the threshold.
  For pyrazinamide: the number of <LLOQs (non-adherent) was 16 out of 79 (20%) for the cases and 46 out of 336 (14%) for the controls across all three visits.
  For rifampicin: the number of <LLOQs (non-adherent) was 41 out of 79 (52%) for the cases group and 152 out of 336 (45%) for the control group across all three visits.
  The results are shown in Table 2. No differences in odds ratio of non-adherence reached statistical significance for either drug, except for pyrazinamide at week 4 (*P* = 0.05). The odds ratio was 3.72 (95% confidence interval [CI]: 1.00–13.9). Boxplots of pyrazinamide and rifampicin concentrations and the proportion of observations that are <LLOQ are depicted in Figure 1.
2. Using personalised thresholds from model-based simulations.

**Table 2.** Frequency table of non-adherence based on method 1 (<LLOQ) and method 2 (personalised thresholds) for pyrazinamide at 24 h for visits on weeks 2, 4, and 8.^**A**^

	Number (%) of samples indicating non-adherence			
	Method 1 (<LLOQ)		Method 2 (2.5th percentile)	Method 2 (5th percentile)
	Rifampicin	Pyrazinamide	Pyrazinamide	
Week 2				
Cases	12/27 (44%)	5/27 (19%)	7/27 (26%)	8/27 (30%)
Controls	49/112 (44%)	12/112 (11%)	15/112 (13%)	15/112 (13%)
Week 4				
Cases	15/26 (58%)	7/26 (27%)	7/26 (27%)	7/26 (27%)
Controls	54/112 (48%)	16/112 (14%)	22/112 (20%)	23/112 (21%)
Week 8				
Cases	14/26 (54%)	4/26 (15%)	5/26 (19%)	6/26 (23%)
Controls	49/112 (44%)	18/112 (16%)	23/112 (21%)	27/112 (24%)
Overall				
Cases	41/79 (52%)	16/79 (20%)	19/79 (24%)	21/79 (27%)
Controls	152/336 (45%)	46/336 (14%)	60/336 (18%)	65/336 (19%)

LLOQ = lower limit of quantification.

A Overall numbers and percentages were calculated based on the total number of all three visits at weeks 2, 4, and 8.

**Figure 2.**
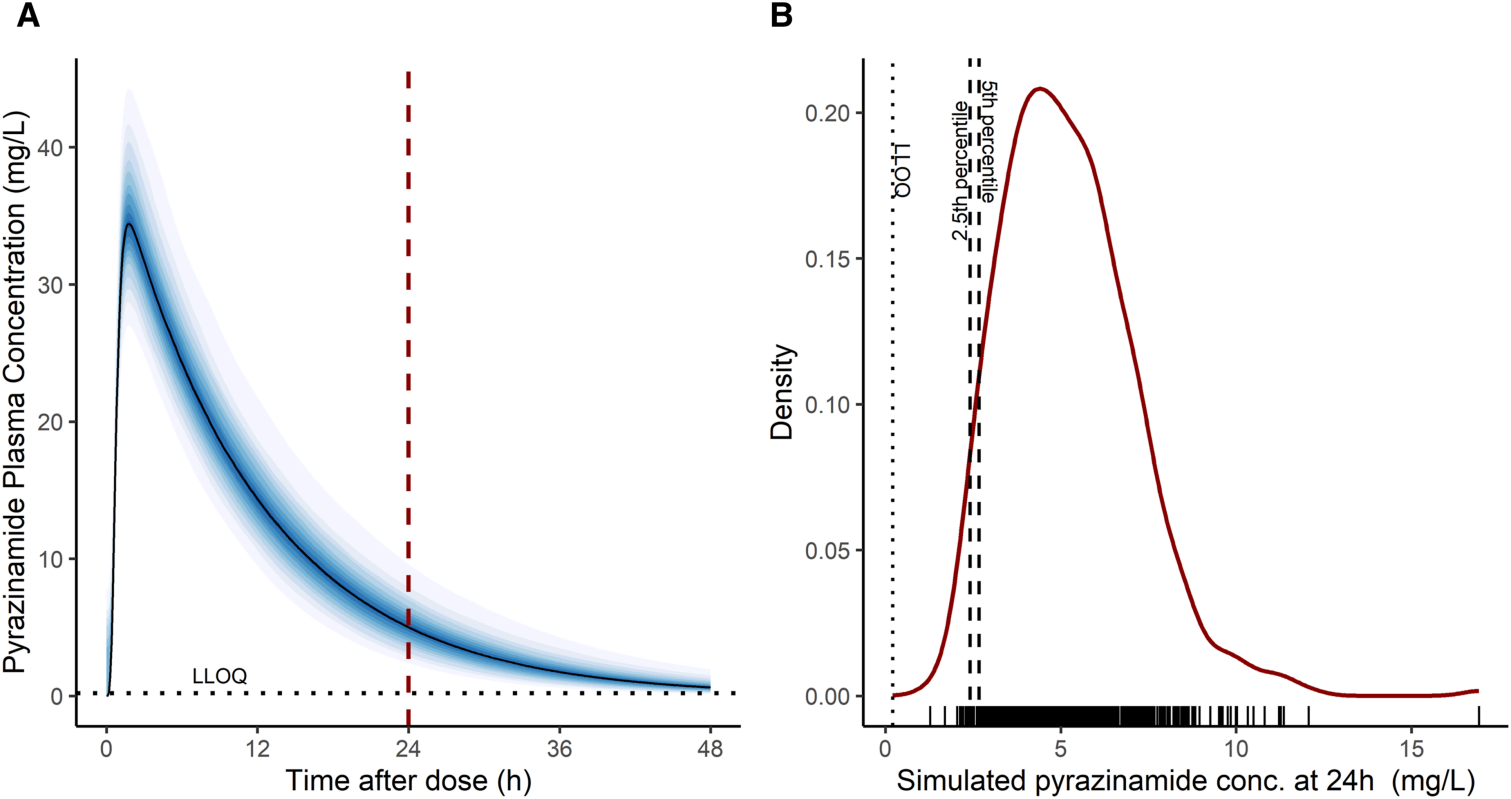
A typical plasma concentration versus time after dose (h) profile (left) and the distribution curve of all 500 simulated concentrations at 24 h for one participant on one visit as an example (right).

At 48 h for pyrazinamide and both 24 and 48 h for rifampicin, the expected drug levels based on the simulations in all participants were below the LLOQ. This means that these timepoints and drugs cannot be used to check whether patients are taking their medication as prescribed. Since the drug levels are expected to be undetectable at these times, whether or not a participant took the medication, any measurement below the detection limit would not provide useful information about adherence. In other words, an undetectable drug level at these points does not necessarily mean the participant missed a dose – it is simply due to drug elimination. As shown in Figure 2, pyrazinamide is expected to be <LLOQ at 48 h. Therefore, the thresholds carried forward to the statistical analysis step were the 2.5th and 5th percentile thresholds for pyrazinamide at 24 h. For the 2.5th percentile, 24% of cases were non-adherent compared to 18% of controls. For the 5th percentile, these proportions were 27% for cases and 19% for controls, respectively. The results are presented in Table 2. An example of a simulated concentration–time profile, as well as the distribution curve of all 500 simulated concentrations at 24 h for one participant on one visit, is depicted in Figure 2.

The results of the conditional logistic regression for both methods are in Table 3. The odds ratio for the 5th percentile at week 2 was the only statistically significant threshold (*P* value = 0.023). The odds ratio was 4.10 (95% CI: 1.21–13.9), indicating that the odds of being non-adherent were 4.10 times higher in the case group compared to the control group. For thresholds other than those at week 8, the results suggested a trend towards higher odds of non-adherence among cases compared to controls, as indicated by odds ratios greater than 1. However, at week 8 (2.5th and 5th percentiles), the odds ratios were less than 1, suggesting a potential trend in the opposite direction. But the confidence intervals for these odds ratios included 1, and the *P* values were >0.05. Therefore, these differences in odds between the cases and controls were not statistically significant.

**Table 3.** Conditional logistic regression results for method 1 (LLOQ threshold) for pyrazinamide and rifampicin, and method 2 (personalised thresholds) for pyrazinamide at 24 h at weeks 2, 4, and 8, adjusting for weight and sex.

Method 1	Pyrazinamide			Rifampicin		
Week	Odds ratio	95% CI	*P* value	Odds ratio	95% CI	*P* value
2	1.75	0.481–6.38	0.39	1.23	0.464–3.27	0.68
4	3.72	1.00–13.9	0.050^**A**^	1.53	0.611–3.84	0.36
8	0.862	0.250–2.97	0.81	1.00	0.397–2.54	0.99

Method 2	Pyrazinamide					
	2.5th percentile			5th percentile		
Week	Odds ratio	95% CI	*P* value	Odds ratio	95% CI	*P* value
2	3.34	0.938–11.9	0.063	4.10	1.21–13.9	0.023^**A**^
4	2.01	0.676–5.96	0.21	1.85	0.634–5.42	0.26
8	0.791	0.250–2.50	0.69	0.769	0.257–2.31	0.64

LLOQ = lower limit of quantification; CI = confidence interval.

A Statistically significant (α = 0.05).

## DISCUSSION

We used two adherence thresholds methods – the LLOQ and personalised model-based simulations – to compare adherence to TPT between participants who developed TB (cases) and those who did not (controls) in the four-drug arm of REMEMBER. Although no significant differences in adherence were observed across all thresholds, statistically significant associations were found for pyrazinamide’s LLOQ threshold at week 4 and the 5th percentile personalised threshold at week 2. The personalised approach was slightly more sensitive. Cases exhibited ∼4 times higher odds of non-adherence than controls. Acknowledging the limitations discussed below, such as unrecorded dosing times, preclude definitive conclusions, our findings hint at a potential but inconclusive trend towards poorer adherence in the cases group that may have contributed to the REMEMBER study’s primary outcome: the four-drug regimen did not reduce mortality at 24 weeks compared to isoniazid-only treatment in outpatient adults with advanced HIV.

One other approach to assess if adherence influenced REMEMBER’s outcomes could have been to evaluate adherence separately within each treatment arm: the four-drug and the isoniazid-only. However, our chosen methodology was ultimately decided by the pharmacokinetic properties of the TB drugs. Due to isoniazid’s short half-life, adherence could not be reliably assessed in the isoniazid-only arm using drug level measurements, that is, isoniazid levels are expected to be undetectable at 24 h regardless of whether the patient is adhering to medication. In the four-drug arm, both pyrazinamide and rifampicin also have relatively short half-lives. We anticipated that pyrazinamide levels would be undetectable after 48 h, and rifampicin levels even sooner, after 24 h. Consequently, we utilised pyrazinamide levels measured at 24 h as an indicator of non-adherence within the four-drug arm. Using the LLOQ as a simple cut-off threshold for non-adherence is considered less sensitive and reliable than using the thresholds derived from our model-based simulations.

There are two limitations to this analysis. The first is that unrecorded times of the dose administration introduce uncertainty into the analysis and influence the model-predicted drug concentration. However, our analysis should be viewed in the context of its exploratory nature; our objective was to apply a modelling methodology to opportunistic data from a study not originally designed for adherence assessment. This is reflective of real-life scenarios where reported times are commonly unavailable or inaccurate. Such analysis could be further improved to yield more accurate results if the times of the last dose were recorded. The second is that adherence was assessed only for the dose taken the day before sampling. If the patient had already taken today’s dose, then it is almost certain that the concentrations will be much larger than a trough, and it will be nearly impossible to detect non-adherence in the previous days (at least for drugs with limited accumulation, like in this case). This restricts the scope of adherence evaluation to a single timepoint, thereby overlooking fluctuations or patterns in adherence behaviour and may provide an incomplete understanding of the participants’ overall adherence. Moreover, the week 8 adherence results should be interpreted with caution, as some participants may have discontinued pyrazinamide before their sample was collected, making the data less reliable. However, a sensitivity analysis excluding these individuals confirmed our findings, showing no significant difference between cases and controls.

## CONCLUSION

Our analysis of the REMEMBER study suggests a trend towards higher non-adherence in cases than controls, though data limitations make this inconclusive. As adherence remains a significant challenge, better monitoring tools are needed. We propose using model-based simulations to establish personalised adherence thresholds – an approach applicable to a wide range of medications beyond TB drugs, including antiretroviral therapy. This method is particularly useful for drugs with long half-lives (e.g., bedaquiline and clofazimine, as shown by Resendiz-Galvan et al.^17^) and is effective in two key scenarios: first, for drugs with low BSV (e.g., lamivudine), where any deviation from expected levels strongly indicates non-adherence,^18^ and second, for drugs with high but predictable BSV based on covariates like genetics (e.g., CYP2B6 genotype for efavirenz) or renal function. For example, if a drug’s pharmacokinetics are largely influenced by renal clearance, individual differences can be accounted for, allowing for a more accurate personalisation of the threshold. In such cases, the observed drug concentration can provide a clear signal of adherence. Moreover, integrating published pharmacokinetic models with simple, low-cost point-of-care assays (e.g., the saliva-based pyrazinamide assay)^19^ to assess whether drug concentrations fall within an expected, model-based personalised range further facilitates this approach, making adherence monitoring more accessible and practical.

## Supplementary Material




