# Supplementary material for: Pharmacokinetic modelling as a tool to assess TB treatment adherence: application to the REMEMBER study
Source: IJTLD Open. 2026 Feb 11;3(2):77–83. doi: 10.5588/ijtldopen.25.0467 (PMC12991563; doi:10.5588/ijtldopen.25.0467)
Supplement: Supplementary file 1 [file ijtldopen25-0467_supplementarydata1.pdf]

Supplementary file: Pharmacokinetic modelling as a tool to assess TB treatment adherence: application to the REMEMBER study

Study population

Participants from the 4-drug arm were included in this sub-study (NWCS440) to assess their adherence. They were administered 4-drug fixed combination tablets (150mg rifampicin, 75mg isoniazid, 400mg pyrazinamide, and 275mg ethambutol) with weight-adjusted daily dosing according to the WHO guidelines13. The participants with confirmed, probable, or clinical diagnosis of pulmonary or extra-pulmonary TB by week 48 (cases) were matched with participants from the 4-drug arm without confirmed, probable, or clinical diagnosis of pulmonary or extra-pulmonary TB by week 48 (controls)4. The case-control matching was done by weight and sex in the ratio of 1:4 (i.e. each case matched with 4 controls) according to the method described by Wang14. Only participants with at least 2 out of 3 blood samples collected at visits on weeks 2, 4, and 8 of the parent study were included.

Drug quantification

After collection, samples were placed in an ice bath, centrifuged in a cooling centrifuge, and then stored at -80°C until the time of analysis. Pyrazinamide and rifampicin plasma concentrations were determined in stored plasma samples by liquid chromatography-tandem mass spectrometry (LC-MS) performed in the Division of Clinical Pharmacology, University of Cape Town [15]. The LLOQs were 0.203 µg/mL and 0.075 µg/mL for pyrazinamide and rifampicin, respectively. Any concentration value below the LLOQ was censored and reported as <LLOQ. The assessment was done based on the exposure of rifampicin and pyrazinamide only; isoniazid and ethambutol were excluded due to their short plasma half-life.

Software

NONMEM® 7.5.0 was used to run the simulations for Method 2. R 4.0.3 was used for data processing, post-processing NONMEM® results and generating figures [19]. The R package "Epi" was used to perform conditional logistic regression tests to calculate the odds ratio.
